# *Mycobacterium tuberculosis* Glucosyl-3-Phosphoglycerate Synthase: Structure of a Key Enzyme in Methylglucose Lipopolysaccharide Biosynthesis

**DOI:** 10.1371/journal.pone.0003748

**Published:** 2008-11-18

**Authors:** Pedro José Barbosa Pereira, Nuno Empadinhas, Luciana Albuquerque, Bebiana Sá-Moura, Milton S. da Costa, Sandra Macedo-Ribeiro

**Affiliations:** 1 Instituto de Biologia Molecular e Celular (IBMC), Universidade do Porto, Porto, Portugal; 2 Centro de Neurociências e Biologia Celular, Departamento de Zoologia, Universidade de Coimbra, Coimbra, Portugal; 3 Departamento de Bioquímica, Universidade de Coimbra, Coimbra, Portugal; Institute of Molecular and Cell Biology, Singapore

## Abstract

Tuberculosis constitutes today a serious threat to human health worldwide, aggravated by the increasing number of identified multi-resistant strains of *Mycobacterium tuberculosis*, its causative agent, as well as by the lack of development of novel mycobactericidal compounds for the last few decades. The increased resilience of this pathogen is due, to a great extent, to its complex, polysaccharide-rich, and unusually impermeable cell wall. The synthesis of this essential structure is still poorly understood despite the fact that enzymes involved in glycosidic bond synthesis represent more than 1% of all *M. tuberculosis* ORFs identified to date. One of them is GpgS, a retaining glycosyltransferase (GT) with low sequence homology to any other GTs of known structure, which has been identified in two species of mycobacteria and shown to be essential for the survival of *M. tuberculosis*. To further understand the biochemical properties of *M. tuberculosis* GpgS, we determined the three-dimensional structure of the apo enzyme, as well as of its ternary complex with UDP and 3-phosphoglycerate, by X-ray crystallography, to a resolution of 2.5 and 2.7 Å, respectively. GpgS, the first enzyme from the newly established GT-81 family to be structurally characterized, displays a dimeric architecture with an overall fold similar to that of other GT-A-type glycosyltransferases. These three-dimensional structures provide a molecular explanation for the enzyme's preference for UDP-containing donor substrates, as well as for its glucose versus mannose discrimination, and uncover the structural determinants for acceptor substrate selectivity. Glycosyltransferases constitute a growing family of enzymes for which structural and mechanistic data urges. The three-dimensional structures of *M. tuberculosis* GpgS now determined provide such data for a novel enzyme family, clearly establishing the molecular determinants for substrate recognition and catalysis, while providing an experimental scaffold for the structure-based rational design of specific inhibitors, which lay the foundation for the development of novel anti-tuberculosis therapies.

## Introduction

Tuberculosis is a re-emerging infectious disease with an increasing prevalence worldwide. There has been no development o f new anti-mycobacterial drugs in the last 30 years and those currently available target a surprisingly small number of essential functions in the cell, mostly related to cell wall biosynthesis [1], [2]. The unique mycobacterial cell wall, characterized by an outer layer of mycolic acids, containing α-ramified β–hydroxylated long-chain fatty acids, is unusually impermeable, and forms a barrier for most common antibiotics [3]. However, the biochemical pathways leading to its synthesis are largely unknown. The complete sequencing of mycobacterial genomes and their subsequent analysis further aided in the identification and characterization of putative enzymes involved in biosynthesis of the complex mycobacterial cell wall structure. In *M. tuberculosis* H37Rv genome analysis revealed that 1% of the ORFs are dedicated to glycosidic bond synthesis, emphasizing the relevance of this group of enzymes for the physiology and pathogenicity of these bacteria [4]. Putative glycosyltransferases (GTs) with a requirement for NDP-sugar donors represent the majority of the classified GTs in *M. tuberculosis*, but most of them have not been characterized biochemically [4].

Glycosyltransferases (GTs) are ubiquitous enzymes that catalyse the transfer of a sugar moiety from an activated sugar donor to saccharide or non-saccharide acceptors, forming glycosidic bonds. Members of the large GT family display extreme diversity in their amino acid sequences, reflecting the large number of different donor and acceptor molecules that are used by this class of enzymes. Sequence similarity and biochemical studies have been used to group the GTs into 91 (as of May 2008) distinct families, GT1 to GT91 [5]. Characteristics of the various families along with current family classification are available from the carbohydrate-active enzyme (CAZy) database (http://afmb.cnrs-mrs.fr/CAZY/). Two main catalytic mechanisms can be displayed by glycosyltransferases: inversion of the anomeric configuration or retention of the anomeric configuration. Despite the large variety of sequence-based GT families and the relatively low sequence homology between them, structural studies revealed that these enzymes fall into two main topologies, the GT-A and the GT-B folds, likely reflecting large constraints in the nucleotide-binding motif [6]–[8]. The difference between the members of these fold families is that GT-A proteins contain a single Rossmann fold domain while GT-B proteins contain two Rossmann fold domains. Curiously both folds have been identified in structures of retaining and inverting transferases, indicating that the fold-family does not dictate the mechanism (for a recent review see [7]).

*Mycobacteria* synthesize unusual polysaccharides containing methylated hexoses [9], namely methylmannose polysaccharide (MMP) and methylglucose lipopolysaccharide (MGLP) [10], [11]. The polysaccharides sequester fatty acyl-CoAs, protecting them from degradation in the cytoplasm, and regulate the activity of fatty acid synthase I [12], [13]. Due to their role in mycobacterial physiology, the innumerous enzymes involved in the biosynthesis of the methylated polysaccharides represent attractive targets for chemotherapy, but most of them remain largely unknown. So far, only an α–(1→4)–mannosyltransferase and a 3–*O*–methyltransferase involved in the synthesis of MMP and a 6–*O*–methyltransferase involved in the synthesis of the MGLP have been characterized [14]–[16]. Glucosylglycerate and diglucosylglycerate have been detected in small amounts in *M. smegmatis* cell extracts and regarded as the putative precursors for MGLP synthesis [17]. Glucosylglycerate (GG) is a versatile molecule, forming the polar head group of a glycolipid in *Nocardia otitidis-caviarum* and existing as a free organic solute in bacteria and archaea, where it accumulates under combined osmotic stress and nitrogen starvation [18], [19]. The related organic solute mannosylglycerate (MG) accumulates in many (hyper)thermophilic bacteria and archaea where it serves as compatible solute against salt stress but has not been detected, so far, in any other biological structure [20]. The most common pathway for the synthesis of MG and GG involves the synthesis of a phosphorylated intermediate, namely glucosyl-3-phosphoglycerate (GPG) or mannosyl-3-phosphoglycerate, from NDP-glucose or NDP–mannose and 3-phosphoglycerate by glucosyl-3-phosphoglycerate synthase (GpgS) or by mannosyl-3-phosphoglycerate synthase. These intermediates are then dephosphorylated by specific phosphatases [21], [22]. *GpgS* homologues have been identified in the genomes of two species of mycobacteria from which the MGLPs have been studied [17], [23], and suggested to be essential for the growth of *M. tuberculosis* H37Rv [1]. Recently, the GpgSs from the fast-growing *M. smegmatis* and from the slow-growing *M. bovis* BCG (identical to Rv1208 from *M. tuberculosis* H37Rv), have been expressed recombinantly and shown to have GpgS activity *in vitro* [24]. The enzymes utilized UDP-glucose (or ADP-glucose) and D-3-phosphoglycerate as substrates, were strictly dependent on Mg^2+^ for catalytic activity, and retained the α-configuration of the donor UDP-glucose.

Mycobacterial GpgSs display low sequence homology with GTs of known structure. Sequence analysis and fold recognition methods predicted that *M. tuberculosis* GpgS should possess a GT-A like fold [25], and it shared a low sequence homology to the spore-coat forming protein SpSA from *Bacillus subtilis* [26], an inverting transferase belonging to the GT2 family. However, enzymatic analysis of *M. bovis* and *M. smegmatis* GpgSs showed that they retain the α-configuration of the UDP-glucose and therefore they were reassigned to the recently created GT81 family [5], [21], [24].

To investigate the structure and specificity of this novel glycosyltransferase family we have solved the crystal structure of *M. tuberculosis* GpgS, both unliganded and in complex with Mg^2+^, UDP and 3-phosphoglycerate. The three-dimensional structure here described constitutes the first representative of the GT81 family, and permits a detailed comparison with other glycosyltransferases of known structure. Furthermore, it allows to elucidate the structural requirements for nucleotide and acceptor recognition and to identify key residues within the active site. Therefore, the three-dimensional structure of *M. tuberculosis* GpgS provides a basis for the rational design of new anti-mycobacterial compounds that might act by inhibiting the initiation of MGLP synthesis, and have potential therapeutic applications.

## Materials and Methods

### Protein expression and purification

*M. tuberculosis gpgS* gene (identical to *M. bovis* BCG *gpgS*) was obtained using chromosomal DNA from *M. bovis* BCG as a template. Recombinant *M. tuberculosis* GpgS was expressed in *E. coli* and purified as previously described [24].

### Crystallization and cryoprotection

Initial crystallization trials were performed at 293K using the hanging-drop vapour diffusion method with the Index sparse matrix crystallization kit (Hampton Research). Approximately 25% of the conditions assayed yielded microcrystals. Single crystals were observed in four different conditions (0.8M potassium sodium tartrate tetrahydrate, 0.1M Tris pH 8.5, 0.5% (w/v) polyethylene glycol monomethyl ether 5000; 0.02M magnesium chloride hexahydrate, 0.1M HEPES pH 7.5, 22% (w/v) Poly(acrylic acid sodium salt) 5100; 0.2M lithium sulfate monohydrate, 0.1M Tris pH 8.5, 35% (w/v) polyethylene glycol 3350; and 0.2M ammonium acetate, 0.1M Tris pH 8.5, 35% (w/v) polyethylene glycol 3350), which were further optimized. The best diffracting GpgS crystals (0.4×0.25×0.25 mm^3^) were obtained within 3 days by mixing 4 µL of protein solution (8mg/mL in 0.01M Tris pH 7.5, 0.001M magnesium chloride) with 2 µL of reservoir solution (0.1M Tris pH 8.0 containing 0.5% (w/v) polyethylene glycol monomethyl ether 5000 and 0.65M potassium sodium tartrate tetrahydrate. Larger crystals could be grown, but they displayed very high mosaicity and diffracted anisotropically to lower resolution.

In order to further understand the nucleotide and sugar specificity of this enzyme family, we have also co-crystallized GpgS with UDP and 3-phosphoglycerate. For this purpose GpgS (0.2mM in 0.01M Tris pH 7.5, 0.001M magnesium chloride) was incubated with UDP (0.2mM) and 3-phosphoglycerate (0.2mM) for 1 hour on ice and then crystallized under the same conditions as the apo enzyme. We have also attempted co-crystallization with UDP-glucose and glucose but the electron density maps for the putative complexes showed no substrates bound to the active site. The extreme mechanical fragility of the crystals hampered soaking experiments with the different substrates, and made the optimization of the cryoconditions instrumental to the quality of the X-ray diffraction patterns. A number of different strategies for cryoprotection were attempted [27], [28], such as quick soaking in mother liquor supplemented with glycerol, MPD or glucose, serial transfer of crystals to mother liquor containing increasing concentrations of the selected cryoprotectant, crystal dehydration [29] prior to flash-cooling and annealing [27]. All these approaches failed to yield well-diffracting crystals, which could only be successfully cryocooled using a combination (10∶5∶5) of well solution, 50% (w/v) sucrose and 60% (v/v) of 500mg/mL NDSB-201 (3-(1-Pyridino)-1-propane sulfonate) in ethyleneglycol (according to the protocol described by A.G Evdokimov at http://www.xtals.org/crystal_cryo.pdf) as cryoprotectant.

### Data collection and processing

X-ray diffraction data sets, extending to 2.5Å resolution for native GpgS and to 2.70 Å resolution for the ternary complex, were collected at 100K at the macromolecular crystallography beamlines ID14EH1 and ID14EH3 (ESRF, Grenoble, France), respectively. The diffraction data were collected using a single crystal in 1° oscillation steps over a range of 180°, and processed using MOSFLM [30] and SCALA from the CCP4 suite [31]. For a summary of data collection statistics see Table 1. The crystals belong to the tetragonal space group I4_1_ and, assuming the presence of one GpgS monomer per asymmetric unit, the Matthews coefficient is 4.57Å^3^ Da^−1^, corresponding to a solvent content of 73.0% [32]. The high solvent content of both native and ternary complex GpgS crystals explains their observed mechanical fragility and the failure of our soaking experiments with different ligands.

**Table 1:** Statistics of data collection and refinement

	Apo GpgS*	GpgS complex*
**Crystallographic analysis**		
X-ray source	ESRF ID14-EH1	ESRF ID14-EH3
Wavelength (Å)	0.934	0.931
Temperature (K)	100	100
Space group	I4_1_	I4_1_
Unit-cell parameters (Å)	a = b = 99.6, c = 126.6	a = b = 100.3, c = 127.0
Solvent content (%)	73.0	73.5
Resolution range (Å)	78.30 - 2.50 (2.64 - 2.50)	70.90-2.70 (2.85-2.70)
No. of reflections (total/unique)	220996/21338 (17962/3117)	54575/16855 (7853/2481)
Multiplicity	10.4 (5.8)	3.2 (3.2)
Completeness (%)	99 (100)	97.6 (98.7)
R_sym_ a	0.070 (0.351)	0.069 (0.330)
R_rim_ b	0.073 (0.386)	0.082 (0.394)
R_pim_ c	0.021 (0.161)	0.042 (0.209)
I/σ (I)	8.3 (2.1)	9.5 (2.6)
**Refinement**		
R_factor_ d/R_free_ e (%)	18.7/22.1	20.4/23.5
N° of unique reflections (working/test set)	20584/1060	1579 6/814
Water molecules	129	86
Ions	0	1 (Mg^2+^)
Total number of atoms	2218	2204
Number of protein atoms	2089	2081
Average overall B-factor (Å^2^)	64.5	45.7
Average protein B-factor (Å^2^)	65.7	45.9
Average main-chain B-factor (Å^2^)	64.5	44.6
Average side-chain B-factor (Å^2^)	67.0	47.3
Average water B-factor (Å^2^)	63.8	40.6
Average ion B-factor (Å^2^)	–	54.5
Average UDP B-factor (Å^2^)	–	41.5
Average 3-Phosphoglycerate B-factor (Å^2^)	–	56.8
r.m.s.d. bonded B's (Å^2^)	3.5	4.0
r.m.s.d. bond lengths (Å)	0.006	0.010
r.m.s.d. bond angles (°)	0.970	1.410
**Ramachandran plot statistics**		
Residues in favoured regions (%)	96.3	94.3
Residues in allowed regions (%)	3.7	5.3
Residues in disallowed region (%)s	0	0.4
**Estimated coordinate error**		
E.s.d. from Luzzati plot (Å)	0.29	0.35
DPI^f^ (Å)	0.18	0.23
Maximal estimated error (Å)	0.30	0.36

a R_sym_ =  ∑*_h_*∑*_i_* |*I_i_*(*h*)−<*I*(*h*)>|/∑*_h_*∑*_i_ I_i_*(*h*), where *I* is the observed intensity and <*I*> is the average intensity of multiple observations of symmetry-related reflections.

b R_rim_ =  ∑*_h_* [*N*/(*N*−1)]^1/2^ ∑*_i_* |*I_i_*(*h*)−<*I*(*h*)>|/∑*_h_*∑*_i_ I_i_*(*h*), where *I* is the observed intensity and <*I*> is the average intensity of multiple observations of symmetry-related reflections.

c R_pim_ =  ∑*_h_* [1/(*N*−1)]^1/2^ ∑*_i_* |*I_i_*(*h*)−<*I*(*h*)>|/∑*_h_*∑*_i_ I_i_*(*h*), where *I* is the observed intensity and <*I*> is the average intensity of multiple observations of symmetry-related reflections.

d R_factor_ = Σ||F_o_|−|F_c_||/Σ |F_o_| where |F_o_| and |F_c_| are observed and calculated structure factor amplitudes, respectively.

e R_free_ is the cross-validation R_factor_ computed for a randomly chosen subset of 5% of the total number of reflections, which were not used during refinement.

f Diffraction-data precision indicator

* Values in parenthesis correspond to the outermost shell.

### Structure solution and refinement

**Figure 1:**
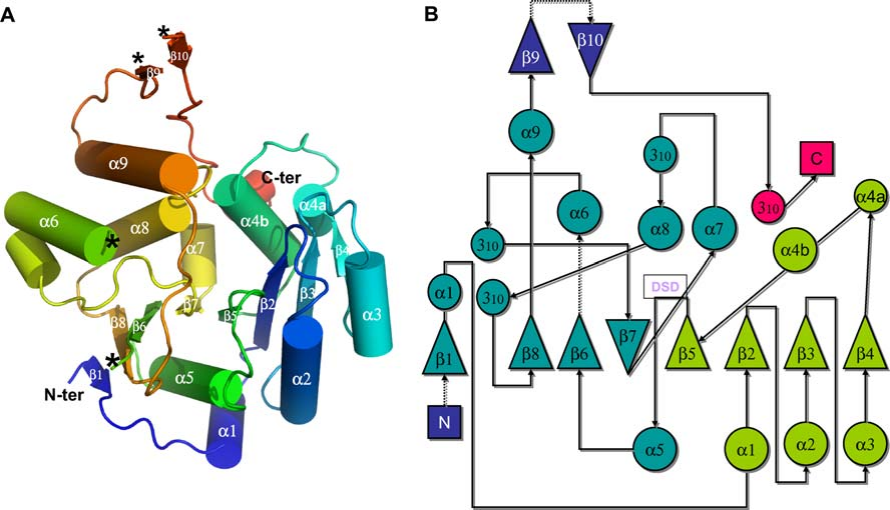
*M. tuberculosis* GpgS structure displays a GT-A fold. a). Cartoon representation of GpgS three-dimensional structure. Helices are represented as cilinders and β-strands are shown as arrows. The structure is coloured from dark blue (N-terminus) to red (C-terminus). Disordered regions at the protein surface that were not visible in the electron density (Arg167-Gly184 and Leu294-Asp302) are indicated by asterisks. b) Topology diagram of *M. tuberculosis* GpgS; the N-terminal Rossmann-like subdomain is coloured green, while the C-terminal subdomain is shown in blue; the conserved Asp-Xaa-Asp (DSD) motif, immediately after strand β5, is boxed; loops that are not visible in the electron density maps are shown as dotted arrows.

**Figure 2:**
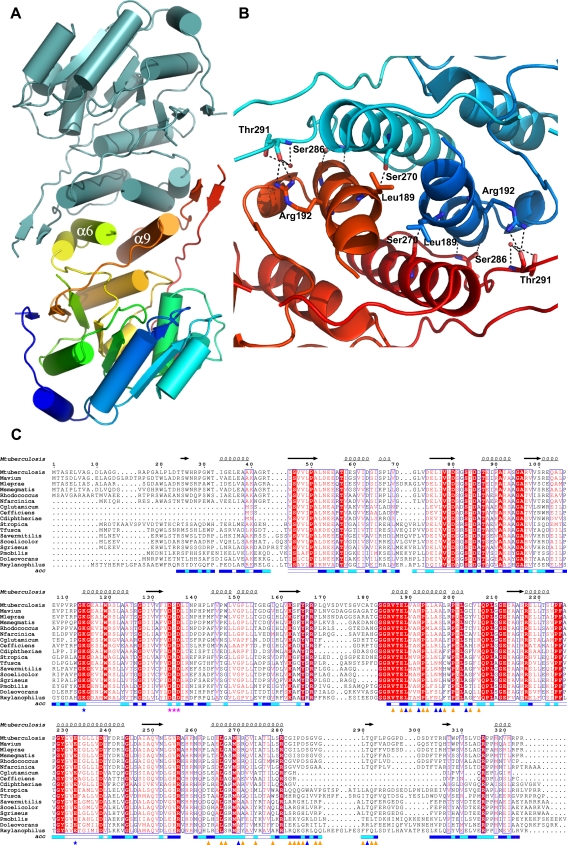
*M. tuberculosis* GpgS forms a tightly packed dimer, through a region conserved amongst homologous mycobacterial enzymes. a) Cartoon representation of the GpgS dimer with the crystallographic twofold axis perpendicular to the plane of the image; helices are represented as cilinders and β-strands are shown as arrows; monomer A is coloured cyan and monomer B is coloured from dark blue (N-terminus) to red (C-terminus); the helices at the dimer interface are labeled. b) Detailed view of the polar interactions formed across the dimer interface. Relevant amino acid residues are represented as sticks (blue for monomer A and red for monomer B) and labelled, and hydrogen bonds are represented by dashed lines. c) Amino acid sequence alignment of the representative mycobacterial GpgSs. The sequence of the homologous enzyme from *R. xylanophilus*, whose structure was used as a phasing model, is also included in the alignment. Identical residues are shown in white with a red background while conserved amino acids are coloured red with a white background. Residues involved in dimerization are indicated by triangles below the alignment, blue if involved in polar interactions and orange if involved in hydrophobic contacts; the conserved Asp-Ser-Asp motif is indicated by pink stars and Lys114 and Glu232, belonging to the Asp134-Lys114-Glu232 conserved ion pair, are indicated by blue stars. The relative accessibility of each residue in the GpgS monomer is represented below the alignment (blue, accessible; cyan, intermediate; white, buried).

**Figure 3:**
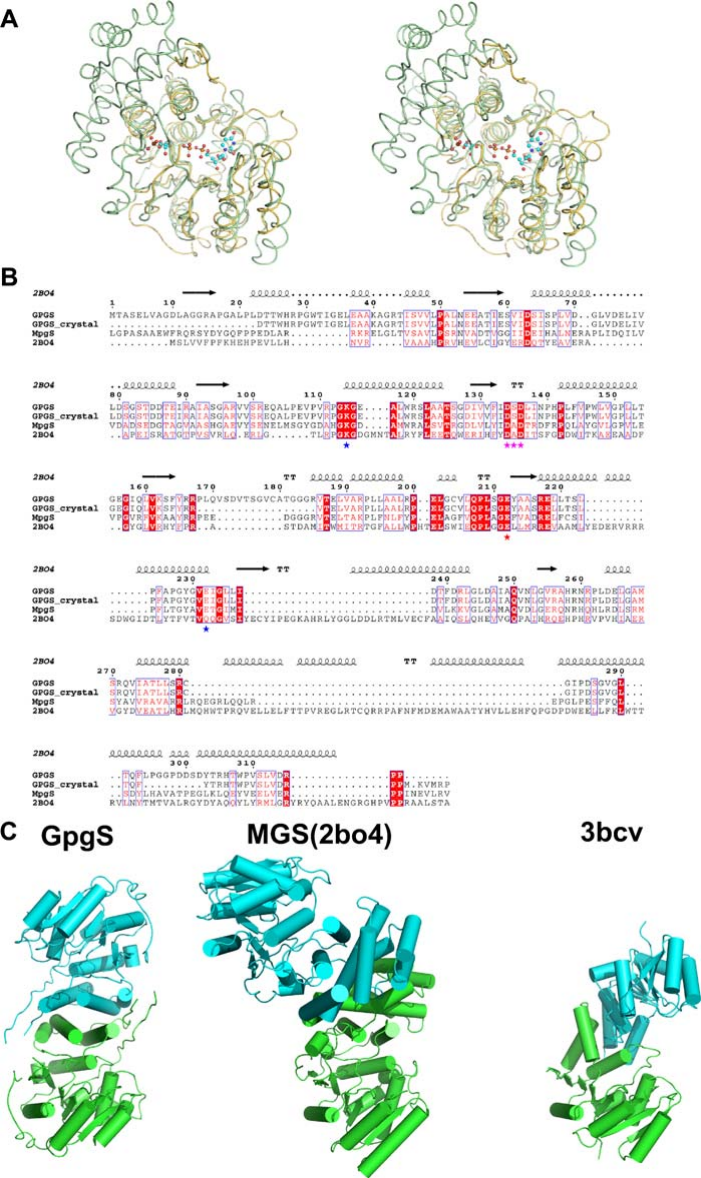
The mycobacterial GpgS shows high structural homology to the mannosylglycerate synthase (MgS) from *Rhodothermus marinus*. a) Stereo representation of the superposed *M. tuberculosis* GpgS (yellow) and *R. marinus* MgS (PDB entry 2BO4, green) three-dimensional structures; the UDP and 3-phosphoglycerate molecules crystallized in complex with GpgS are shown in a ball-and-stick representation (carbon, cyan; oxygen, red; nitrogen, blue; phosphorous, orange). b) Structure based sequence alignment of GpgS (this work), *R. xylanophilus* MpgS [34], and *R. marinus* MgS [43]. For clarity, the sequence of the crystallographic GpgS model (with the missing N-terminal region and 166–183 loop) was also included in the alignment (GpgS-crystal). The secondary structure of MgS is represented above and conserved residues are indicated by stars below the alignment. c) Cartoon representation of the dimer interface of structurally homologous glycosyltransferases. From left to right: GpgS from *M. tuberculosis*; MgS from *R. marinus* (PDB entry 2BO4); putative GT from *B. fragilis* (PDB entry 3BCD).

**Figure 4:**
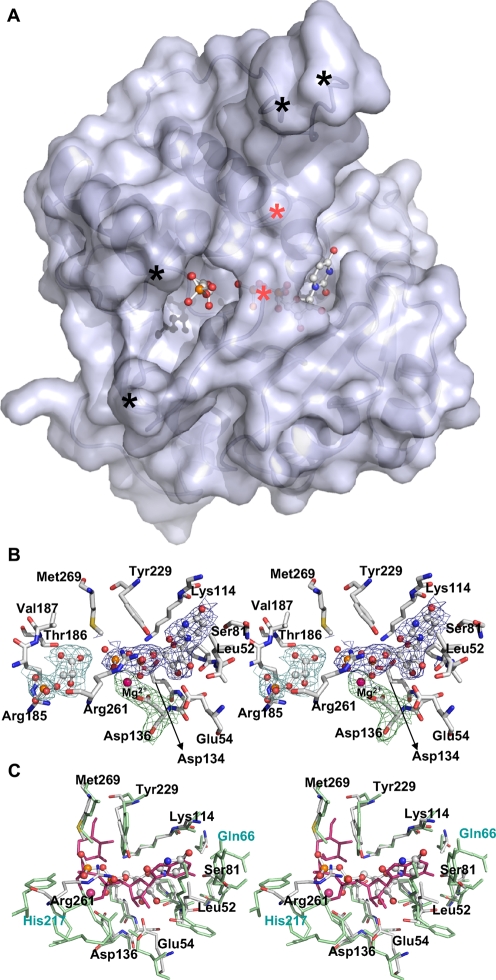
The UDP binding pocket forms a rigid framework that is conserved in structurally homologous GT's. a) Surface representation of the GpgS structure highlighting the perfect fit of UDP and 3-phosphoglycerate to the enzyme's active site. The bound UDP (on the right) and 3-phosphoglycerate (on the left) are shown as ball-and-stick models. b) Stereo-view of the UDP binding site showing the 2Fo-Fc map calculated at 2.7 Å and contoured at 1σ around the ligands. Mg^2+^, UDP and 3-phosphoglycerate are represented as ball-and-stick models, and the amino acid residues forming the nucleotide binding pocket are represented as sticks and labeled. c) Stereo view of the superposed nucleotide binding pockets of GpgS (grey) and MgS (green). The UDP molecule is represented as a ball-and-stick model, and the GDP-mannose bound to MgS (PDB entry 2BO8) is shown in red. The residues forming the binding pocket are represented as sticks and labeled (black for GpgS and green for MgS residues). Colour-code: carbon, light grey; oxygen, red; sulfur, yellow; magnesium, pink; nitrogen, blue; phosphorous, orange).

**Figure 5:**
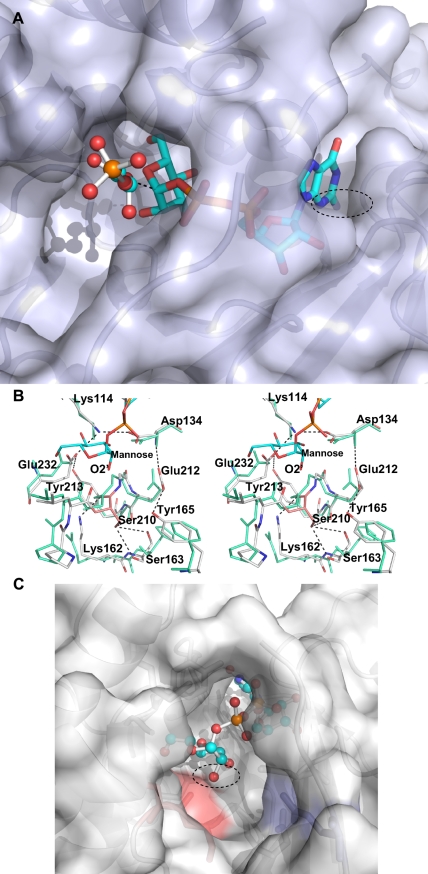
GpgS acceptor and donor sugar binding pockets. a) Close view of the modeled GpgS complex with GDP-mannose and 3-phosphoglycerate. The GpgS active site is shown as a surface representation, the 3-phosphoglycerate is shown as a ball-and-stick model (colour-coded as in Fig. 4), and the GDP-glucose is shown in yellow sticks. A dashed circle is drawn around the GDP NH2 group that cannot be accommodated within the nucleotide binding pocket, due to the orientation of the side chain of Ser81 (see Fig. 4). A dashed line between the oxygen of the acceptor 3-phosphoglycerate and the anomeric C1 of the mannose is shown corresponding to a distance of 2.8 Å. b) Detailed stereoview of the GpgS (colour coded as in Fig. 4) donor sugar binding site in comparison with MgS (green). Some of the GpgS residues mentioned in the text are labeled, and hydrogen bonds are represented by dashed lines. The mannose moiety is shown at the top of the image in blue sticks and Ser210 is highlighted in salmon. c) View of the acceptor sugar binding pocket indicating that a movement of the region containing the amino acids Leu209 and Ser210 would be necessary to prevent steric clash with the O2 of mannose.

Initial phases were determined by molecular replacement with the program Phaser [33] using the partially refined structure of the homologous MpgS/GpgS (PDB entry 3F1Y) from *R. xylanophilus* [34] as search model. Initial model building was performed with ARP*/*wARP [35] and was followed by iterative cycles of manual building with Coot [36], refinement with REFMAC5 [37] and in the final stages with phenix.refine [38]. Water molecules were added automatically and checked with phenix.refine [38] and Coot [36]. The geometry restraint information for refinement of the ligands (UDP and 3-phosphoglycerate) was determined with phenix.elbow [38]. The stereochemical quality of the final model was assessed with the programs MOLPROBITY [39] and PROCHECK [40], and agreement between the atomic model and X-ray data was verified with SFCHECK [41]. Refinement statistics are shown in Table 1. Coordinates and structure factors were deposited in the RCSB Protein Data Bank under accession numbers 3E25 (ternary complex) and 3E26 (apo GpgS). Figures 1a, 2a, 2b, 3a, 3c, 4a, 4b, 4c, 5a, 5b, and 5c were prepared with PyMol (http://pymol.sourceforge.net). Figures 2c and 3b were prepared with ESPript [42].

## Results and Discussion

### Overall structure of *M. tuberculosis* GpgS

*M. tuberculosis* GpgS crystallizes as a monomer in the asymmetric unit and its structure was solved by molecular replacement using the partial three-dimensional model of the homologous (37% identity) *R. xylanophilus* MpgS/GpgS (GenBank accession code EU847586) [24], [34], and refined to a crystallographic R-factor of 18.7% (R-free = 22.1%) at 2.5 Å resolution with good stereochemical parameters (Table 1). The model could be readily traced with the exception of the first 22 amino acid residues and two surface loops encompassing residues Arg167-Gly184 and Leu294-Asp302. Those flexible regions were not unambiguously interpretable in the final electron density maps and were thus omitted from the final model.

The structure of *M. tuberculosis* GpgS comprises a single globular domain with an overall conical shape, displaying an eight-stranded, predominantly parallel β/α/β sandwich (Fig. 1a). The topology of the central β-sheet is β4, β3, β2, β5, β7, β6, β8, β1 with strand β7 running in the anti-parallel direction (Fig. 1b), and it displays similarities with the previously described GT-A fold-containing GTs [26], [43]. The structure can be subdivided into two closely associated subdomains, the N-terminal nucleotide binding subdomain (residues 45–137), composed of a four stranded β-sheet (β4, β3, β2, β5) flanked on either side by two α-helices and resembling the Rossmann-like fold, and the C-terminal subdomain (residues 138–281), consisting of a mixture of α/β secondary structure, usually highly variable in GTs with the GT-A fold, and shown to be involved in acceptor substrate recognition [6]. A type I β-turn (Asp134-Leu137) at the C-terminal end of strand β5 delimits the N-terminal subdomain, and contains the conserved Asp-Xaa-Asp (Xaa, any amino acid) motif implicated in divalent metal ion coordination (see below).

Overall, this GpgS, as happens for most GTs, has a low secondary structure to loop ratio, and most of the flexible loops seem to be involved in substrate recognition and binding [26], [44]. In the structure presented here, the large non-conserved C-terminal region comprising residues Cys281 to Pro323, although containing a short hairpin and a one turn 3_10_ helix, is mostly devoid of regular secondary structure (Fig. 1a). This region does not have any structural counterpart in other structurally-characterized enzymes with the GT-A fold, and forms a large solvent exposed loop, external to the main core of the structure, that covers helix α9 and folds back into the nucleotide binding sub-domain inserting between helices α4b and α7. The functional significance of these extra C-terminal regions remains undetermined. A closer look at the crystal packing surfaces shows that the C-terminal surface regions are engaged in numerous crystal contacts, namely the C-terminal 3_10_-helix is in close proximity to helix α3 of the neighboring molecule and the long loop containing the short β-hairpin (β9–β10) forms both polar and van der Waals contacts with the C-terminal portion of helix α6 across the crystallographic two-fold axis (Fig. 2a). Particularly, a detailed analysis shows a tight packing of the GpgS molecules related by crystallographic twofold symmetry, accompanied by the formation of an extensive “dimer” interface provided by interactions between the crystallographically-related helices α6 and α9 and maintained by both polar and hydrophobic interactions (Table 2, Fig. 2b). An amino acid sequence alignment of mycobacterial GpgSs shows that the regions involved in formation of the putative dimer are very well conserved (Fig. 2c). Namely, residues Leu189, Pro193 and Leu266, involved in hydrophobic interactions across the potential dimer interface are strictly conserved (Table 2). The potential involvement of this interface in protein oligomerization in solution was accessed with the Protein Interfaces, Surfaces and Assemblies Server (PISA [45]). Indeed, “dimerization” is accompanied by burial of 9.4% of each monomer's total accessible surface (1332.2Å^2^). The extension of this interaction surface suggests that the dimer seen in the crystal is related to the oligomeric GpgS species observed by size exclusion chromatography [24]. The elongated nature of the putative *M. tuberculosis* GpgS dimer, with overall dimensions of 85×31×39 Å^3^, might explain its higher apparent molecular weight in size exclusion chromatography [24]. Taken together, these data indicate that *M. tuberculosis* GpgS is a dimer in solution and that homodimerization might be a conserved feature within this enzyme family.

**Table 2:** Summary of interactions at the twofold axis dimer interface

Molecule A	Distance (Å)	Molecule B
**Hydrogen bonds**		
**Arg192 (NH1)**	3.0	Thr291 (O)
**Arg192 (NH2)**	3.0	Thr291 (O)
Ser270 (OG)	2.5	**Leu189 (O)**
Ser286 (N)	2.8	Ala197 (O)
Ser286 (OG)	2.6	Ala196 (O)
Thr291 (OG1)	3.8	Gly203 (O)
Thr291 (O)	3.0	**Arg192 (NH1)**
Thr291 (O)	3.0	**Arg192 (NH2)**
**Leu189 (O)**	2.5	Ser270 (OG)
Ala197 (O)	2.8	Ser286 (N)
Ala196 (O)	2.6	Ser286 (OG)
Gly203 (O)	3.8	Thr291 (OG1)
**Side chain hydrophobic interactions**		
**Leu189 (CD2)**	3.7	**Leu266 (CB)**
Val190 (CG2)	3.7	Val190 (CG2)
**Pro193 (CB)**	3.6	Ile274 (CD1)
Ala197 (CB)	3.7	Arg271 (CZ)
**Pro200 (CG)**	3.8	Ser286 (CB)
**Pro200 (CB)**	3.7	Val288 (CG2)
Leu206 (CD2)	3.3	Phe293 (CD1)
Leu206 (CD2)	3.5	Phe293 (CG)
Leu206 (CD2)	3.5	Phe293 (CB)
Ile283 (CD1)	3.7	Ile283 (CG2)

**Bold** - strictly conserved residues

### Structural Similarity to Known Glycosyltransferases

A DALI search [46] for structurally similar proteins showed that the closest structural homologues of *M. tuberculosis* GpgS belong to the glycosyltransferase superfamily and display the GT-A fold. Its closest structural homologue is the bacterial mannosylglycerate synthase from *Rhodothermus marinus* [43], showing an overall rmsd of 2.2 Å, over 216 structurally equivalent Cα atoms (Fig. 3a,b). Interestingly, the following similarity matches, with Z-scores higher than 13.5 and rmsd's around 3.0–3.5Å, are to the catalytic domains of human retaining glycosyltransferases belonging to the GT27 family, such as UDP-GalNAc:Polypeptide α-N-Acetylgalactosaminyltransferase-2 and UDP-GalNAc:Polypeptide α-N-acetylgalactosaminyltransferase (pp-GalNAc-T10), suggesting a distant evolutionary relationship between these proteins. Such structural homology was not apparent from the primary sequence data or from other approaches such as threading or fold recognition programs that take into account secondary structure information [25]. Indeed, the sequence-based alignments indicated that *M. tuberculosis* GpgS closest homologue with a known structure was the pore-coat forming protein SpSA from *Bacillus subtilis* [26] (180 aligned Cα atoms, with a rmsd of 3.5Å), an inverting transferase belonging to the GT2 family. Other distantly related structural homologues with the GT-A architecture are the bacterial nucleotidyl transferases (Table 3) [47], reflecting a strong conservation of the nucleotide binding architecture. The additional subdomains are normally associated with selectivity towards the sugar donor and acceptor and are often involved in protein oligomerization. It is noteworthy that some of the structural homologues of *M. tuberculosis* GpgS are also oligomeric, such as the mannosylglycerate synthase (MgS) from *Rhodothermus marinus* (PDB entry 2BO4) [43] and the putative GT from *Bacteroides fragilis* (PDB entry 3BCV) (Fig. 3c). The latter is also predicted to be a dimer by protein interaction interface analysis [45] and, although the residues involved in homodimerization are not conserved, an interaction between α-helices also mediates dimerization (Fig. 3c).

**Table 3:** *M. tuberculosis* GpgS structural neighbours

PDB ID	Brief description	Rmsd(Å)	Z-score (identical overlapping residues)
3f1y	Mannosyl-3-phosphoglycerate synthase (retaining GT81)	1.7	36.3 (37%)
2bo8	Mannosylglycerate synthase (retaining GT78)	2.2	21.0 (23%)
2ffu	Polypeptide N-acetylgalactosaminetransferase 2 (retaining GT27)	3.4	14.5 (13%)
2oi6	Bifunctional protein GLMU	3.1	13.7 (13%)
2d7r	Polypeptide N-acetylgalactosaminetransferase 10 (retaining GT27)	3.4	13.7 (15%)
3bcv	Putative glycosyltransferase protein (inverting GT2)	3.1	13.6 (18%)
1h7t	3-deoxy-manno-octulosonate cytidylyltransferase	3.1	13.5 (16%)
1g97	N-acetylglucosamine-1-phosphate	3.2	13.2 (11%)
1qgq	SpsA –spore coat polysaccharide biosynthesis protein (inverting GT2)	3.6	13.1 (11%)
1hv9	UDP-N-Acetylglucosamine pyrophosphorylase	3.1	13.1 (13%)

### Nucleotide binding pocket

*M. tuberculosis* GpgS catalyzes the formation of glucosyl-phosphoglycerate, using an activated glucose donor and 3-phosphoglycerate [24]. The enzyme displays a strong preference for UDP-glucose, although ADP–glucose is also an efficient donor. GDP–glucose could be used albeit with very low efficiency. In order to further understand the nucleotide and sugar specificity of this enzyme family, we have succeeded in obtaining the structure of the GpgS-UDP-phosphoglycerate ternary complex at 2.7 Å resolution (Fig. 4a) and the ligands UDP, 3-phosphoglycerate and Mg^2+^ could be readily located in the electron density maps (Fig. 4b). The structure of the ternary complex was refined to a final R-factor of 20.4% (R-free 23.5%), and binding of the nucleotide does not induce gross structural changes in GpgS (overall rmsd 0.28 Å over 279 equivalent Cα atoms). The only significant structural changes occur in the loop connecting strand β8 and helix α9, where large movements of Asn260 and Arg256 are observed. The flexibility of this loop impaired modeling of the Ala257-His258-Arg259 segment in the ternary complex. The occupancy of the UDP and Mg^2+^ ion in the final structure is below unity, indicating that not all active sites are occupied by these ligands. Consequently, the loop containing residues Ala257-His258-Arg259, as well as residues that are found to interact with the bound nucleotide and probably with the metal ion (see below), might have more than one conformation in the crystals of the ternary complex, thus explaining its apparent disorder upon ligand binding.

The GpgS active site resides between the two subdomains (Fig. 1b), and while the Rossmann-like subdomain provides a rigid framework for nucleoside binding, movement of the loop connecting strand β8 with helix α9 is observed in order to accommodate the phosphate groups of the ligand (Fig. 4a). Although with fractional occupancy in our crystal structure, the electron density clearly indicates that the UDP molecule binds in a hydrophobic cleft located in the Rossmann-like N-terminal subdomain of GpgS (Figs. 1a and 4a, b). The top wall is defined by the main chain of the conserved residues Pro112-Gly113-Lys114 and by the aliphatic portion of the side chain of Lys114, while the bottom wall of the nucleotide binding cavity is defined by the side chain of the also conserved Leu52 (Fig. 4b), as well as by the Pro50-Ala51 segment at the back and by the side chain of Ser81, maintained in position by formation of an hydrogen bond between its main chain carbonyl and the side chain of Arg101 (replacing the guanine binding residue Gln66 of *Rhodothermus marinus* MgS)[43]. The uracyl moiety makes hydrogen bonds to the side chain of Tyr229 (Trp189 in the homologous MgS), although the electron density in this region indicates that it could also rotate and hydrogen bond to the side chain of Ser81.

A detailed comparison of the structural features that define the nucleotide base binding sites in GpgS and MgS (Fig. 4c) reveals a number of relevant amino acid substitutions that might explain the stronger preference of GpgS towards uracyl and its ability to utilize ADP- but not GDP-sugar donors [24]. The nucleotide base binding cavity in GpgS is similar in size to the one in MgS, but the position of residue Ser81 in GpgS (Tyr87 in MgS), would prevent binding of the NH_2_ at position 2 of the guanine moiety due to steric hindrance (Fig. 5a). Since the remaining binding pocket-forming residues are conserved, a shift in position of the purine base would not be feasible. MgS accepts both GDP- and UDP-linked sugars, although with a strong preference for the purine nucleotide. This specificity can be attributed to the presence of Gln66 in MgS at the loop linking strand β4 to helix α4b, replaced by Arg101 in GpgS (within the 3_10_ helix α4a). Arg101 lies further away from the substrate binding pocket due to the different conformation of this loop in both structures (Fig. 3a). In order to understand how GpgS can also utilize ADP-sugar donors [24], we have also modeled an ADP molecule into the GpgS active site. This purine base, lacking the NH_2_ at position 2, could be positioned in this pocket without clashing with the side chain of Ser81 (data not shown).

In contrast, the residues that interact with the ribose and phosphate moieties of the nucleotide are well conserved in GpgS and MgS. Both ribose hydroxyls (O2 and O3) form hydrogen bonds to Glu54 (Glu11 in MgS) and the O3 interacts also with the main chain amide of Ser135 (from the conserved Asp^134^Xaa^135^Asp^136^ motif). The first aspartate of the Asp-Xaa-Asp motif does not bind the nucleotide, but forms instead an hydrogen-bonded network with Lys114 and Glu232 (Lys 76 and Asp192 in MgS), strictly conserved in MgS and in other retaining GTs such as the well studied galactosyltransferase LgtC from *Neisseria meningitidis* (PDB entry 1GA8) [48]. The second aspartate coordinates a Mg^2+^ ion that bridges the oxygens of the UDP α and β phosphates. The Mg^2+^ ion displays axial coordination to the oxygens (O1B and O1A) of the α- and β-phosphate groups and to Asp136 (OD2). In the homologous MgS structure a manganese ion is found at this position coordinated by Asp102 (equivalent to Asp136 in GpgS), His217, and the phosphates of the bound nucleotide. Residue His258, located in the loop connecting strand β8 and helix α9, is in the apo-enzyme in a structurally equivalent position to His217 in apo-MgS. In the ternary complex of GpgS the electron density around this region is un-interpretable, probably because in some molecules the His side chain might be facing the coordinated metal and in other it might shift its position by 180°, as observed in the structures of MgS with and without bound nucleotide [43]. The phosphate moiety in GpgS is further stabilized by polar interactions between the α-phosphate and the side chain of Tyr 229 (Trp189 in MgS, no interaction with bound nucleotide), and between the α- and β-phosphate and the side chain of Arg261 (structurally close to Tyr 220 in MgS, no interaction with bound nucleotide).

### Sugar donor binding pocket

Biochemical data show that GpgS strongly discriminates against mannose as a sugar donor [24]. In order to understand the structural determinants for sugar donor specificity, and profiting from the high structural homology between GpgS and MgS (Figs. 3a and 3b), a model of GpgS in complex with GDP-mannose was created by superimposing the structure of the GpgS ternary complex with the MgS-GDP-mannose complex (PDB entry 2BO8, [43]) using the DaliLite server [46]. The residues forming the donor sugar binding pocket are well conserved in both GTs. The pocket is formed by Leu209, Met269 and Tyr 229 (Leu163, Met229 and Trp189 in MgS) on one side (Fig. 4c), and the Asp134-Lys114-Glu232 hydrogen-bonded network on the other side (Figs. 4c and 5b). The insertion of the donor sugar at this position would allow the formation of hydrogen bonds between Glu232 (Asp192 in MgS) and the O6 and O4 oxygens of the glucose/mannose moiety, while the side chains of Asp134 and Lys114 would provide polar contacts with the O3 oxygen, as observed for the structurally equivalent Asp192, Asp100 and Lys76 in MgS. The sole difference between mannose and glucose is the position of the O2 oxygen that is axial in mannose and equatorial in glucose. If we model mannose into this position, with the O2 of the sugar in the axial position, it would then be too close to the carbonyl group of Leu209 (within the conserved PL**S**GE motif, PL**G**GE in MgS). The tight fit of the sugar moiety into this pocket would prevent a shift in the position of the mannose moiety, in order to prevent steric hindrance with this carbonyl group. Although the PLSGE loop is approximately in the same position in GpgS and MgS, the presence of a glycine residue in MgS would facilitate slight movements in the protein structure to better accommodate this substrate. This movement is prevented in the GpgS structure by the presence of a serine residue (Ser210) that is involved in an hydrogen bond network to the main chain atoms of Gln207, Glu212 and Ser163, and the side chain of Ser163 (His127 in MgS). Further stabilization of this loop is provided by contacts between the side chains of Tyr165 (Phe129 in MgS) and Glu212, and between Tyr213 (Leu167 in MgS) and Glu232. A movement of this loop would imply the disruption of numerous hydrogen bonds, making mannose binding energetically unfavorable. As no steric hindrance can be predicted upon binding of the O2 epimer glucose, the presence of a hydrogen-bonded network involving stabilization of the PL**S**GE motif could be indicative of discrimination against mannose as a donor substrate in this family of enzymes.

### Acceptor binding pocket

The catalytic reaction involves the recognition of both donor and acceptor moieties by suitable domains. In contrast to what is observed for the more exposed phosphate moiety, disordered in our ternary complex (Fig. 4b), there is clear electron density for the carboxylate region of the bound 3-phosphoglycerate, which forms hydrogen bonds with the main chain nitrogen of Val186 (Ile138 in MgS) and with the side chain of Thr187 (Thr139 in MgS) (Fig. 4b), closely resembling the interactions identified in the MgS-D-glycerate complex [43]. Further interactions of the 3-phosphoglycerate molecule with GpgS are likely established with residues from the disordered loop connecting β-strand β6 and α-helix α6 (Fig. 1), for which we found no interpretable electron density. In this position, the hydroxyl group from the bound acceptor is 2.8 Å away from the anomeric C1 of the modeled acceptor sugar (Fig. 5a), properly positioned for the glycosyl transfer reaction to occur.

### Implications for catalysis

The enzymes catalyzing glycosyl group transfer are classified as inverting or retaining depending on the stereochemistry of the newly formed glycosidic bond (for a recent review see [7]). The structural analysis of retaining and inverting GTs has demonstrated that there are no specific structural features that determine the stereochemical outcome of the reactions catalyzed by these enzymes. The inverting GTs catalyze the reaction by a direct displacement S_N_2-like mechanism, involving an active site residue that acts as a base catalyst, usually a Glu or Asp located within hydrogen bonding distance to the hydroxyl group of the acceptor. Structural characterization of retaining GTs has failed to present any convincing conservation of structural features on the β-face of the donor sugar substrate and an unifying proposal for their mechanism of reaction. The only strictly conserved structural feature on the β-face of the sugar donor binding site is the Asp134-Lys114-Glu232 ion pair network. Two possible reaction mechanisms have been proposed based on the structural analysis of retaining GTs: The first one involves the formation of a covalently bound glycosyl-enzyme intermediate and requires the positioning of a nucleophile in the vicinity of the anomeric carbon from the sugar donor and located on its β-face; an alternative mechanism proposes the formation of a short-lived ion pair intermediate, where the leaving phosphate group is adequately positioned for acting as a base catalyst.

Analysis of the modeled GpgS-3-phosphoglycerate structure in complex with the sugar-donor (UDP-glucose), indicates that there are no side chains in the proximity of the sugar anomeric C1 carbon that could act as base catalysts. The side chain of Glu232, interacting in our model with the O6 and O4 oxygens of the sugar donor, is structurally equivalent to the conserved base catalyst that activates the acceptor in inverting GTs (e.g Asp191 in *B. subtillis* SpsA [26]), although located distantly from the acceptor nucleophile in our structure. As observed for MgS [43], the only protein atom located on the β−face of the sugar donor and in the proximity (4.0 Å) of the anomeric carbon is the Leu208 main chain carbonyl (Fig. 5b, Leu163 in MgS). This carbonyl might be implicated in stabilizing the cationic intermediate during catalysis [7]. The conserved Asp134-Lys114-Glu232 ion pair network, together with the Mg^2+^ ion, likely play a role in facilitating departure of the leaving phosphate group.

The determination of the three-dimensional structure of *M. tuberculosis* GpgS, unliganded and in complex with UDP and 3-phosphoglycerate, allowed us to identify the crucial residues for nucleotide discrimination and for aceptor recognition. The determination of the structure of this novel mycobacterial drug target will now allow the targeting of specific residues that could impair its function. Furthermore, this structure will provide the framework for structure-based design of novel GpgS inhibitors that could lead to the development of novel anti-mycobacterial compounds.
